# Authors’ response to the letter entitled ‘zero value-added tax on fruit and vegetables: beyond health and fiscal standards’

**DOI:** 10.1017/S1368980024001629

**Published:** 2024-09-24

**Authors:** Luc L Hagenaars, Tera L Fazzino, Joreintje D Mackenbach

**Affiliations:** 1 Department of Public and Occupatonal Health, Amsterdam UMC location University of Amsterdam, Amsterdam, The Netherlands; 2 Department of Psychology, University of Kansas, Lawrence, KS, USA; 3 Cofrin Logan Center for Addiction Research and Treatment, University of Kansas, Lawrence, KS, USA; 4 Epidemiology and Data Science, Amsterdam UMC location Vrije Universiteit Amsterdam, Amsterdam, The Netherlands; 5 Upstream Team, Amsterdam, The Netherlands

In response to our commentary on the Dutch attempt to reduce the value-added tax (VAT) on fruits and vegetables to zero percent [author name] highlighted the importance of expanding perspectives on this issue to planetary health. We appreciate [author name’s] insights and would like to emphasise our agreement with their overall message. It is indeed crucial to address public health nutrition challenges from a planetary health perspective, as compellingly argued by the EAT-Lancet commission^(1)^. According to this commission, to safely feed 10 billion people by 2050 within planetary boundaries, food systems must evolve. This will require a dietary shift to more vegetables, fruits, whole grains, legumes, nuts, unsaturated oils, fewer red meat, processed meat, added sugar, refined grains and starchy vegetables. Like most dietary patterns that are good for human health^(2)^, the EAT-Lancet diet is more expensive that current diets and making it affordable to the world’s poor would require higher incomes, nutritional assistance and lower food prices^(3)^.

Our commentary on reducing VAT on fruits and vegetables tells a story about the political and administrative realities of formulating policies that might contribute to this dietary shift. The observed institutional friction to such policy in the Netherlands underscores the different priorities and languages spoken by health and fiscal experts with formal roles in government advise. Health and fiscal experts were observed to differ in their disciplinary agendas, goals, policy experiences and criteria for ‘good’ evidence. We further argue how the lack of integration between healthy food classifications hinders both groups from reaching common ground and that this confuses political decision-makers. This makes food policy an even harder area than it already is, a point also acknowledged by [author name] regarding the frequent omission of food intake in climate change policymaking.

[Author name’s] letter raises a pertinent question about the policymaking dynamics that might emerge if climate change and animal welfare were included in the formulation of fiscal public health nutrition policies. Based on our observations in the Netherlands, we hypothesise two possible scenarios. The first scenario involves increased institutional friction due to additional demarcation challenges. Policymakers would need to consider not only healthiness classifications but also varying perspectives on what constitutes food that is beneficial for human health, the planet and animal welfare. A recent German analysis showed that adapting the German VAT system towards a zero rate for organic vegetarian food and raising VAT for conventional meat and fish to 19 % would avoid 5·3 billion Euros in external climate costs^(4)^. Yet, deciding which foods should get a tax break and which ones should make up for lost revenue, and getting public and political support for these decisions, would become more complex as the criteria for these decisions expand, and as new experts with other priorities and languages get engaged.

The alternative and more optimistic scenario is that incorporating animal welfare and, especially, climate change, could help facilitate a shift in which parts of government are responsible for food policy. There are currently few economic incentives and legal benchmarks for food policy to improve human health since food policy is predominantly shaped by agriculture, trade and economic policy departments whose institutional goals often focus on maximising sales^(5)^. A planetary health argumentation might gain more traction because the Paris Climate Agreement imposes legal obligations on governments to achieve specified climate goals. There is an objective belief that ‘Paris’ contributes to significant progress in promoting renewable energy, but ‘Paris’ has thus far failed to deliver concrete changes to food systems, even when climate experts agree that changing food systems is necessary for addressing climate change^(6,7)^. As global temperatures rise, pressure on governments to address food policy might build, eventually elevating food policy to a higher agenda position and away from policy venues whose main goal is short-term economic growth. In this hypothetical scenario, institutional friction towards public health nutrition policies might be overcome, which process might look similar to how the Framework Convention on Tobacco Control was established^(8)^.

As [author name] rightfully notes, an interdisciplinary approach is necessary for a comprehensive analysis of public health nutrition. In our view, this should involve not just fiscal and health aspects, nor merely elements related to climate change and animal welfare, but also political and social sciences that seek to understand the ‘black box’ of policymaking in the real world. We hope that the discussion our commentary has triggered contributes to this type of knowledge.
